# Globin ferryl species: what is the nature of the protonation event at pH < 5?

**DOI:** 10.1007/s00775-024-02089-3

**Published:** 2024-12-19

**Authors:** Cezara Zagrean-Tuza, Lavinia Padurean, Maria Lehene, Adrian M. V. Branzanic, Radu Silaghi-Dumitrescu

**Affiliations:** Department of Chemistry, Babes-Bolyai University, 11 Arany Janos Str., 400028 Cluj-Napoca, Romania

**Keywords:** Globin, Heme, Ferryl, Protonation, UV–Vis, DFT

## Abstract

The ferryl state in globins has previously been reported to undergo a protonation event below pH 5, as assessed using pH jump experiments with stopped-flow UV–Vis spectroscopy. This protonation entails hypsochromic shifts in the α and β bands (~ 20 to 40 nm) and an ~ 10 nm reduction in the energy difference between these two bands. We now report that in Mb this event is also characterized by a hypsochromic shift in the Soret band (~ 5 nm). No similar shifts in Soret, α, and β bands are seen upon the denaturation of ferryl Mb with guanidine—suggesting that the spectroscopic changes in ferryl Mb at pH < 5 are not caused by changes in the solvent exposure or in hydrogen bonding around the ferryl unit. Under the same denaturing conditions (pH jump below pH 5, and/or guanidine), ferric-aqua and ferrous-oxy Mb show no spectral changes of the order seen in the ferryl pH jump experiments. Together, these observations suggest that the protonation event is localized on the iron-bound oxygen atom, as opposed to somewhere on a hydrogen-bonding partner. Time-dependent density functional theory (TD-DFT) calculations were not able to systematically predict the UV–Vis spectra of the heme to the level of detail needed to interpret the experimental findings in this study.

**Graphical abstract:**

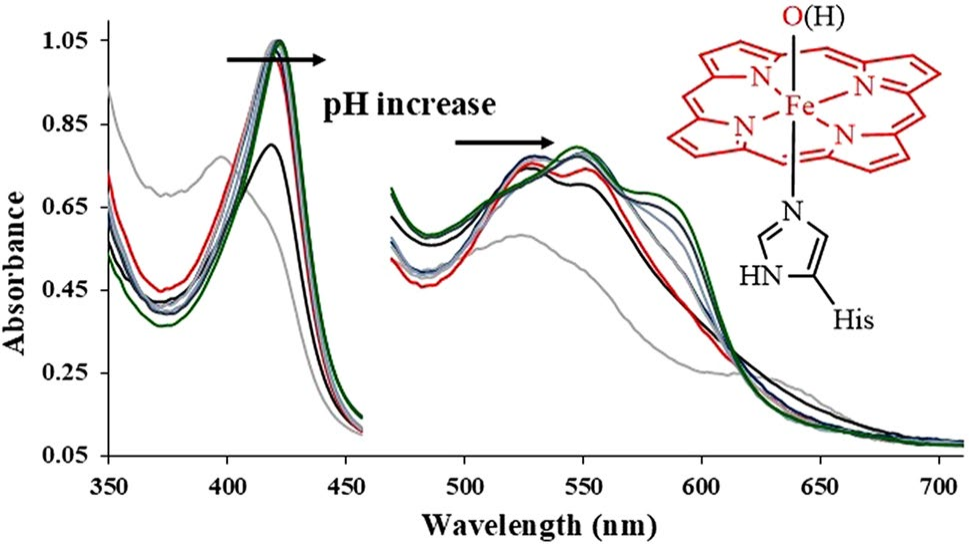

**Supplementary Information:**

The online version contains supplementary material available at 10.1007/s00775-024-02089-3.

## Introduction

Oxygen–oxygen bond cleavage in ferric-hydroperoxo complexes is a common theme in heme-containing proteins and especially in enzymes [1–8]. In heme-containing peroxidases (where the protein-derived “proximal” axial ligand is a histidine imidazole), distal-site acid–base catalysis by His and Arg residues is deemed essential for cleaving the O–O bond within the ferric-hydroperoxo intermediates. The distal pocket of cytochromes P450 (where the protein-derived “proximal” axial ligand is a cysteine thiolate) contains no such residues [9]. Many other important differences in reactivity between peroxidases/globins and cytochromes P450 stem from the nature of the proximal aminoacid. For example, the P450 catalytic cycle entails the reduction of the ferrous-oxy (redox isomer of ferric-superoxo) complex to ferric-peroxo, while histidine-ligated proteins appear generally unable to perform this reaction [9]. Moreover, the Compound I of cytochromes P450 is able to much more efficiently activate C–H bonds, compared to the peroxidase Compound I. The enzyme heme oxygenase (HO) offers an interesting mix of properties: it features a coordination sphere similar to that of globins, and yet it engages in a catalytic cycle resembling the thiolate-ligated P450 [7, 9–16].

**Fig. 1:**
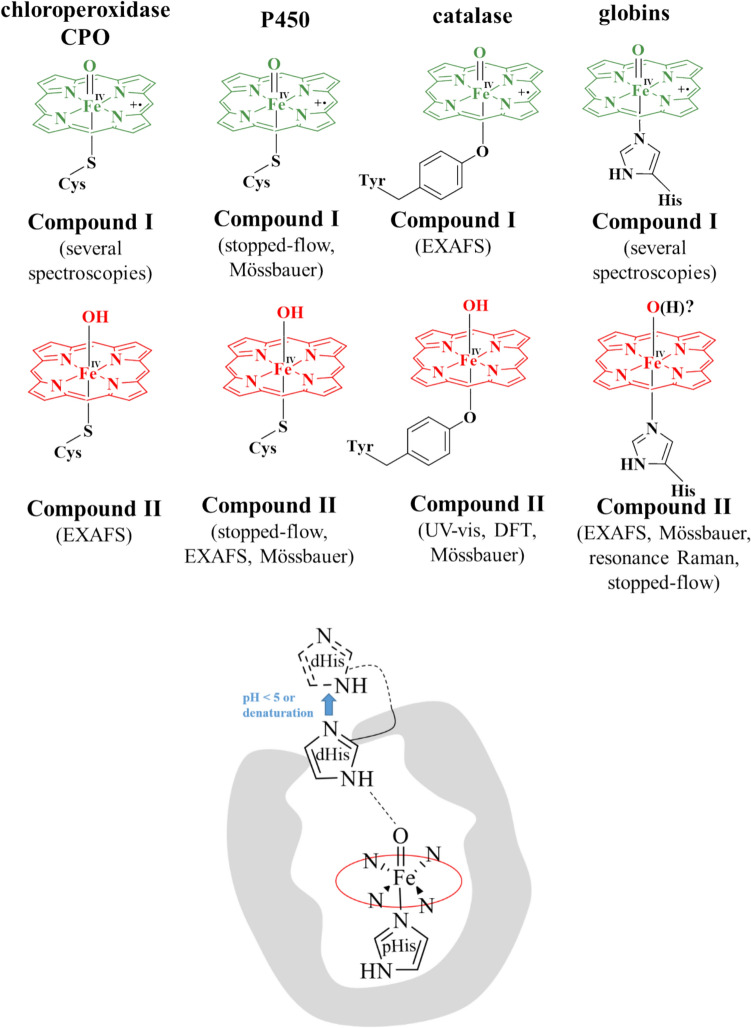
Top: summary of general consensus regarding ferryl protonation in hemoproteins. Bottom: schematic illustration of the possible role of the distal histidine (dHis) in globins at acidic pH, as a possible alternative interpretation of the protonation event on ferryl globins below pH = 5

The thiolate vs. imidazole difference in heme proteins also implies, importantly, that the charge of Compound I (see Fig. 1) is higher by one unit in peroxidases vs. cytochromes P450, rendering the former a stronger oxidant. The same trend would then be followed by their one-electron reduced counterpart, Compound II (see Fig. 1). The redox potentials measured or computed for high-valent ferryl species do support this line of reasoning [4, 5, 17–21]. The basicity of the oxygen atom may also be affected by the thiolate vs. imidazole difference in axial ligation. Indeed, the protonation state of the ferryl unit in the Compound I and Compound II species of hemoproteins has been reconsidered over the past ~ 20 years (see Fig. 1 for a current summary). Due primarily to Green’s work, it is now generally accepted that thiolate-ligated but not histidine-ligated Compound II species are protonated [19–22]. There is evidence that supports the protonation of the histidine-ligated Compound II in peroxidases and globins, but it is disputed [4, 23–27]. One argument against a protonated histidine-ligated ferryl stems from the relatively long Fe-OH bond (~ 1.9 Å) observed in some peroxidase or globin crystal structures assigned as protonated Compound II Fe(IV)-OH. According to DFT calculations and model compounds, a typical Fe(IV)-OH distance is ~ 1.7 to 1.8 Å, ~ 0.15 Å shorter than the corresponding Fe(III)-hydroxo state and longer compared to the deprotonated ferryl. This computed distance may lengthen when hydrogen bonding to the ferryl oxygen is taken into account. In a limiting structure, with a Fe(IV)-aqua heme center, the Fe–O bond length was indeed computed to be ~ 2.05 Å; if so, then the 1.8–1.9 Å Fe(IV)-OH distances observed experimentally may be assigned to very strong hydrogen bonding. In addition, the partial deprotonation of the axial histidine (as seen in heme peroxidases but not in globins) is expected to elongate the Fe(IV)-OH bond only minimally (up to 0.01 Å) [4]. Based on these considerations, protonated ferryls with 1.8 or even 1.9 Å Fe-OH bonds cannot be ruled out entirely.

A second argument against the protonated ferryl has entailed the spectral properties of the proposed protonated ferryl in peroxidases—and more specifically whether structures with differing Fe(IV)-OH bonds may display indistinguishable UV–vis spectra. Various spectral data have been reported on crystals assigned as containing protonated ferryl based on X-ray diffraction, to support the fact that these crystals had not undergone autoreduction to ferric-hydroxide/aqua/penta-coordinated states [4, 23–27]. However, these were contradicted by the spectral data on (frozen) solutions, interpreted as due to non-protonated ferryl—and hence implying that any protonation event down to pH 3 would only be due to the protein (e.g., the distal His) [19–21]. The room-temperature UV–Vis spectra of ferryl globins have on the other hand been interpreted to reveal protonation below pH 5, with the spectra of Fe(IV)=O and Fe(IV)–OH very similar to each other, but still distinguishable [27, 28]. The above considerations are summarized in Fig. 1.

Reported here are the UV–Vis measurements (including stopped-flow) on ferryl myoglobin (Mb) over a wide pH range (2–10), revealing for the first time the Soret band of this species at pH < 5. Comparisons between the behavior of ferryl and that of both met and oxy Mb under the same conditions are provided. Also, to deconvolute the possible contributions of protein denaturation below pH = 5, control experiments are performed with an alternative denaturing agent – guanidine (Gu). The detailed efforts to rationalize the differences in spectra between ferryl Mb at acidic vs. neutral/basic pH are described.

## Materials and methods

Horse heart myoglobin (Mb) was purchased from Sigma Aldrich (Munich, Germany). Myoglobin was dissolved in ultrapure water to a final concentration of 2.5 mM, treated with a 1.5 excess potassium ferricyanide, and then passed through a desalting column (PD-10 desalting column, Cytiva) to ensure a homogenous metmyoglobin stock solution. Hydrogen peroxide 30% was purchased from ChemPUR and diluted accordingly. Where needed, guanidine hydrochloride was added to reaction mixtures from a 3 M stock solution (dissolved in 10 mM borate buffer pH 9). Universal buffer (40 mM phosphoric acid, 40 mM acetic acid, and 40 mM boric acid, adjusted with sodium hydroxide) was used for the pH jump experiments.

UV–Vis spectra were measured using a Lambda 25 (PerkinElmer Singapore) spectrophotometer. The stopped-flow spectra were collected on a Biologic SFM-300 system equipped with three syringes and sequential mixing, with a high-speed diode array detector. The stopped-flow data was processed with the SPECFIT32 software package (BioLogic system, Claix, France). All experiments were performed at room temperature (22 °C).

To produce ferrylmyoglobin, a 20 µM solution of met Mb in 10 mM borate buffer pH 9 was mixed with a 1.5-fold excess of H_2_O_2_ (30 μM). After ~ 40 min, the met Mb was converted into ferryl Mb (more than 95%). The ferrylmyoglobin was stable throughout the pH jump experiment. The Compound II thus obtained was titrated using the pH jump experiments within the pH range 2–10. The final concentration of the ferrylmyoglobin solution after mixing with the buffer solutions was 6.7 µM. Similar pH jump experiments were performed with met Mb (15 µM stock solution in 10 mM pH 9 borate buffer, final concentration after mixing—5 µM), and oxy Mb (9 µM stock solution in 10 mM pH 7 phosphate buffer, final concentration of the protein after mixing—3 µM). The spectral data were collected starting 2 ms after mixing; unless otherwise specified, the spectra reported in the Figures are averages over the first 48 ms after mixing.

To observe the spectral changes induced by the increase in protein mobility, guanidine (Gu) was used as a chaotropic agent. Thus, a Mb solution (final concentrations: 3 µM oxy, or 5 µM met, or 5 µM ferryl- prepared with a 1.5-fold excess of hydrogen peroxide) was mixed with a Gu stock solution (final concentrations of the Gu as mentioned in Fig. 3; the pH of each buffer solution used was adjusted in the presence of 3 M guanidine). The UV–vis spectra were collected 20 min after mixing.

Calculations on the met-aqua heme were performed using a model consisting of a ferric porphyrin ligated axially by an imidazole and with all lateral substituents of the heme replaced by hydrogen (total charge + 1). Trans to the imidazole, axial ligands were placed as indicated in the text. All calculations are performed in vacuum unless otherwise specified; solvated calculations were performed using the CPCM solvation model. The Gaussian software package was employed [29]. Graphical representations of the computed spectra were generated using the Chemcraft graphical interface [30], with Lorentzian broadening and a coefficient of 50.

## Results

### Ferryl UV–Vis in pH jump experiments

**Fig. 2:**
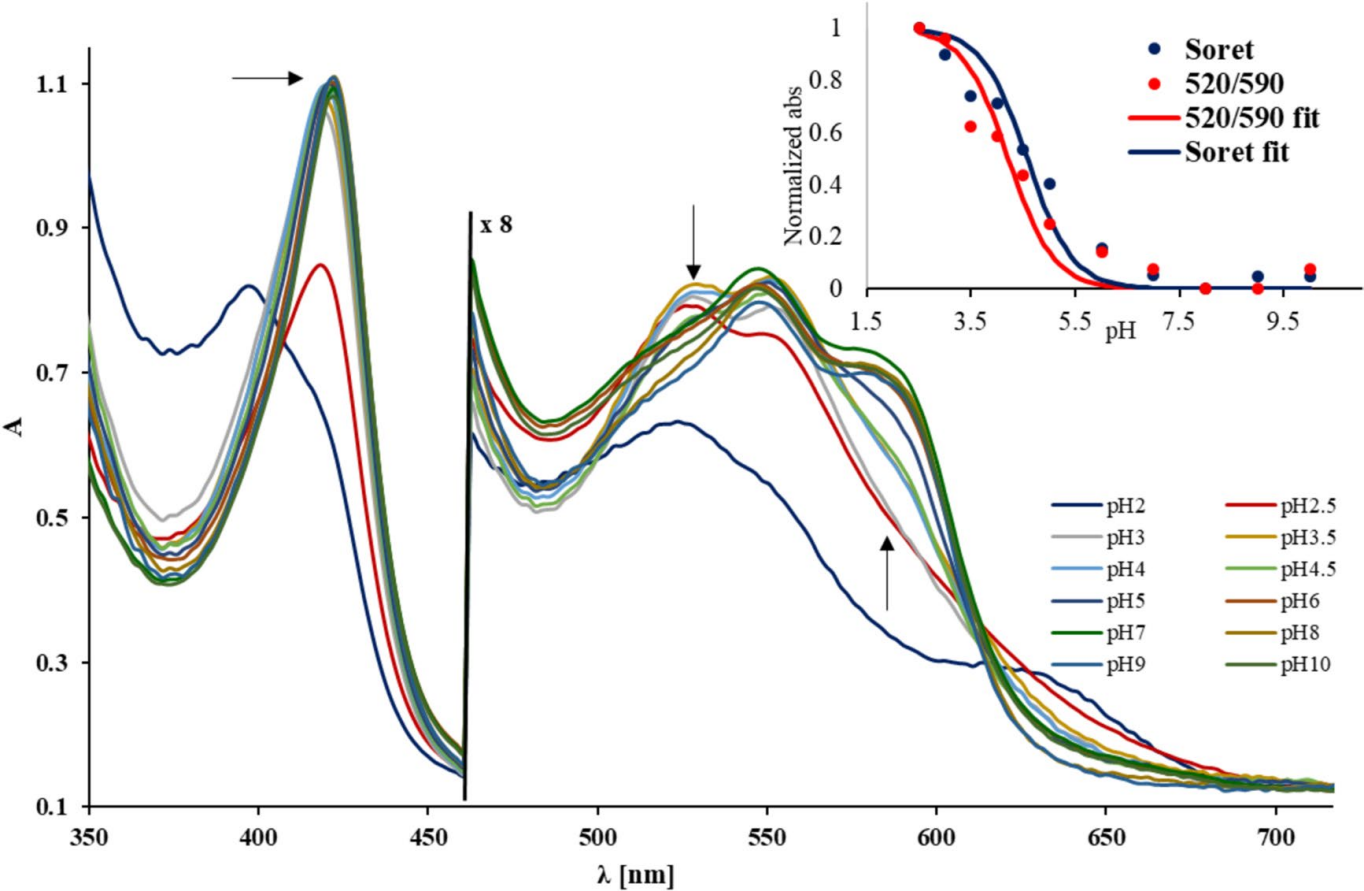
UV–Vis spectra were collected in a stopped-flow experiment titrating 6.7 μM ferryl Mb (final concentration after mixing) against 120 mM universal buffer of pH values as indicated in the legend, at room temperature. The insert shows the titration curves of the ferryl Mb determined from the pH-jump experiment. The curves are built using the wavelengths from the Soret region (in blue- average between the absorbance at 418 nm and 420 nm) and from the α and β region (in red, wavelength ratio as shown). The pKa calculated using the Soret-derived curve is 4.6 (4.58), while the pKa determined based on the changes in the α and β is 4.2

**Fig. 3:**
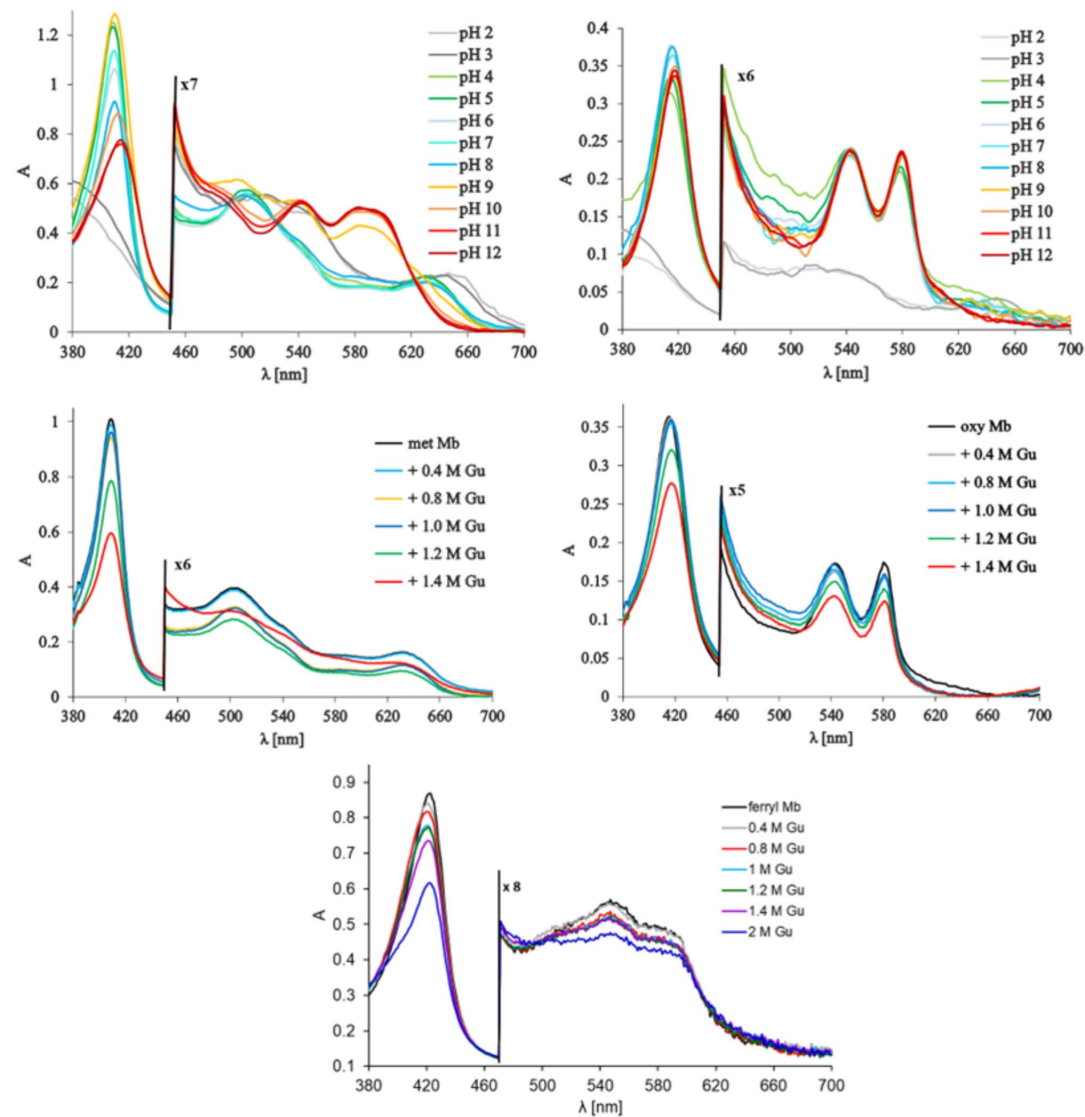
First row: UV–Vis spectra obtained in a stopped-flow experiment mixing met Mb (5 µM final concentration after mixing in 10 mM borate, pH 9, left panel) or oxy Mb (3 µM final concentration after mixing in 10 mM phosphate pH 7, right panel) against 120 mM universal buffer solutions at pH values as indicated in the legend, at room temperature. Second row: UV–vis spectra of met Mb (5 µM final concentration, 20 mM phosphate pH 7, left panel) and oxy Mb (3 µM final concentration in 20 mM borate, pH 9, right panel) at various guanidine concentrations as indicated in the legend, 20 min after mixing, room temperature). Third row: UV–Vis spectra of 5 µM ferryl Mb (final concentration, prepared with a 1.5 H_2_O_2_ excess, in 20 mM borate pH 9) at various guanidine concentrations as indicated in the legend, 20 min after mixing, room temperature

Figure 2 reiterates the previously-reported UV–vis data on ferryl Mb obtained from the pH jump experiments, and now enhances them with newly-collected data in the Soret region. We thus find that the pH ~ 5 transition of ferrylMb entails a change of only ~ 5 nm in the Soret band, as opposed to 20–40 nm in the α and β bands. Such a small change is in fact susceptible to secondary events such as partial protein denaturation. However, such effects can be ruled out based on experiments carried out with the met and oxy Mb, where such shifts in the Soret band were not observed upon pH titration (vide infra, Fig. 3). The pK_a_ calculated based on the Soret region is 4.6 (by applying the Henderson–Hasselbach equation on the ratio between the 418 nm and 422 nm absorbances), which is in reasonable agreement with the pK_a_ determined from the α and β region (4.2 in Fig. 2, or 4.6 in reference [27]). We have previously [27] used the large change in the α and β bands as an argument for a protonation event (concerning the Fe–O bond in ferryl Mb) as opposed to a simple change in hydrogen bonding upon acidification of ferryl Mb. The much smaller change in the Soret band now puts the previous conclusion in a different light.

### Comparison with met and oxy Mb and denaturation experiments

To further understand the changes in Fig. 2, a number of control experiments were performed as illustrated in Fig. 3. The ferric-aqua (met) form of Mb is known to feature a water ligand in contact with the distal His. The denaturation of met Mb at acidic pH entails the movement of the distal His away from the heme and from the water ligand, leaving the distal site solvent exposed. As seen in the pH-jump data (Fig. 3), this change in the environment has a negligible effect on the UV–Vis spectrum (except for pH 2, where complete denaturation already occurs very early on the stopped-flow time scale). Thus, the interaction of the distal His with the heme does not have a major effect on the UV–Vis spectrum in the ferric case at acidic pH (whereas, as it is well known, above pH 8 a clear transition is seen from the spectrum of the high-spin met aqua to the low-spin met hydroxo). The same insensitivity to pH is observed for oxy Mb even below pH 5, as also shown in Fig. 3—even though it is well established that the distal His is hydrogen-bonded to the O_2_ ligand. Figure 3 also shows the effect of guanidine (Gu) as a denaturing agent on the spectra of ferryl, met, and oxy Mb. Similarly to the acidic pH, guanidine is known to denature the protein and enhance solvent accessibility, thus modifying the hydrophilicity/polarity of the heme site. The Mb spectra of all three species (ferryl, met, oxy) show only minor changes in the presence of up to 2 M Gu (a concentration large enough to perturb the tertiary structure), distinctly smaller than the 20–40 nm shifts seen in the pH jump spectra of the ferryl Mb. Overall, the data in Figs. 2 and 3 suggests that a notable protonation event occurs upon acidification of ferryl, but neither upon acidification of met or of oxy Mb nor upon the simple denaturation of the protein. Hence, this protonation event (and the spectral change thereafter) is intrinsic to the ferryl unit rather than to the protein in general.

### Computational predictions on the spectral properties of ferryl protonation

Subtle variations in the UV–Vis spectra of hemoproteins and model complexes can offer insight into the electronic structure and reactivity, and have often served as grounds for controversies as in the case of globin ferryl protonation [19, 21, 27, 31–33]. Major events such as protein unfolding or ligand exchange typically entail significant spectral changes, though exceptions are also known [34–36]. The denaturation of a heme protein is such a major event; in globins, the denaturation leads to a distinct bathochromic shift and a clear widening and hypochromism of the Soret band. These changes have been interpreted as signs of heme deligation and its subsequent aggregation—either in stacked μ-oxo oligomers or in globules of mostly denatured protein.

The simulation of the hemoprotein UV–Vis spectra using TD-DFT and related calculations [37–41] may be useful for differentiating between the UV–Vis spectra of protonated vs. non-protonated ferryl units. To explore this, we analyzed a set of reference systems as detailed in Supporting Information Figures S1-S6. For the S = 5/2 met (ferric) aqua form of histidine-ligated heme, relevant for globins and other histidine-ligated hemoproteins, a number of functionals and solvation models were tested. Most of the functionals were able to predict a Soret band close to 400 nm for the S = 5/2 ferric-aqua heme model, but always ~ 50 nm (~ 0.4 eV) lower than the experiment. The best-performing functional was M06-2x/def2SVP. Nevertheless, even in this case, the agreement with the experiment was only semi-quantitative, as the Soret and β bands (the two most intense in the visible region of the spectrum) are both predicted to be shifted by ~ 50 nm (~ 0.2 eV). The only functional able to simulate some reasonable semblance of the α and β bands was M06-2x; the next best choices, TPSS and M06L, predict a single band in the 500–600 nm region, instead of the two well-separated bands observed experimentally. None of the other functionals/methods predict a band of any sort reminiscent of the α and β. The M06-2X/def2-SVP approach was subsequently employed for monitoring the effect of axial ligand distortions and of the dielectric effects on the spectrum of the ferric-aqua heme. Although solvation did switch the Soret band by ~ 20 nm (~ 0.2 eV) compared to vacuum, variation of the dielectric constant in the 4–80 range had no notable effect on the predicted UV–Vis spectra. The distortions on the axial ligands (water or imidazole) were further explored cf. Figure S4. The rotation of the ligands or lateral displacement appears to have no notable effects. The elongation of the axial ligands leads to hypsochromic and hyperchromic shifts in the Soret band and the opposite effect in the α and β bands; the energy/wavelength difference between these two latter bands did not change significantly. None of these computed changes matches those seen experimentally in ferryl Mb upon protonation below pH = 5 (i.e., hypsochromic shifts in all three bands, and a smaller energy difference between the α and β bands).

The data in Figure S1 suggests that the M06-2X would be the only functional to predict a heme spectrum that would include 3 bands in the visible region similarly to the experiment for a model of the S = 5/2 ferric-aqua state of globins [though still ~ 50 nm off from the experiment (~ 0.4 eV for the Soret band, ~ 0.2 eV for the 630 band)]. However, as shown in Supporting Information Figure S5, the very same methodology fails on a model of the S = 1/2 ferric cyano complex of globins: no α or β bands are predicted at all. Figure S7 shows that the same problems arise for the related S = 1/2 ferric-hydroxo model.

Together, the tests on the three ferric reference models, aqua, cyano, and hydroxo suggest that not one single TD-DFT method can qualitatively predict UV–vis spectra of heme complexes in general. Instead, agreement with the experiment is only possible on a very narrow and closely related set of models using any given functional. Indeed, here, the S = 1/2 hydroxo and cyano-ferric heme-histidine models behave very similarly to each other—but then very different from the S = 5/2 ferric aqua. Thus, a change in spin state and/or overall charge /protonation state may be enough to dramatically change the performance of a functional. Figures S7-S9 show that the Fe(IV)-oxo and Fe(IV)-hydroxo models suffer from the same problems as the S = 1/2 models, in that the α and the β bands cannot be modeled in a meaningful comparative way between the two protonation states. Figure S9 also shows that the inclusion of elements that reduce the porphyrin symmetry (i.e., lateral substituents, and even structural distortions from planarity copied from the crystal structure) do not lead to predicted TD-DFT spectra that would include the α and the β bands in both the Fe(IV)-oxo and Fe(IV)-hydroxo models.

**Fig. 4:**
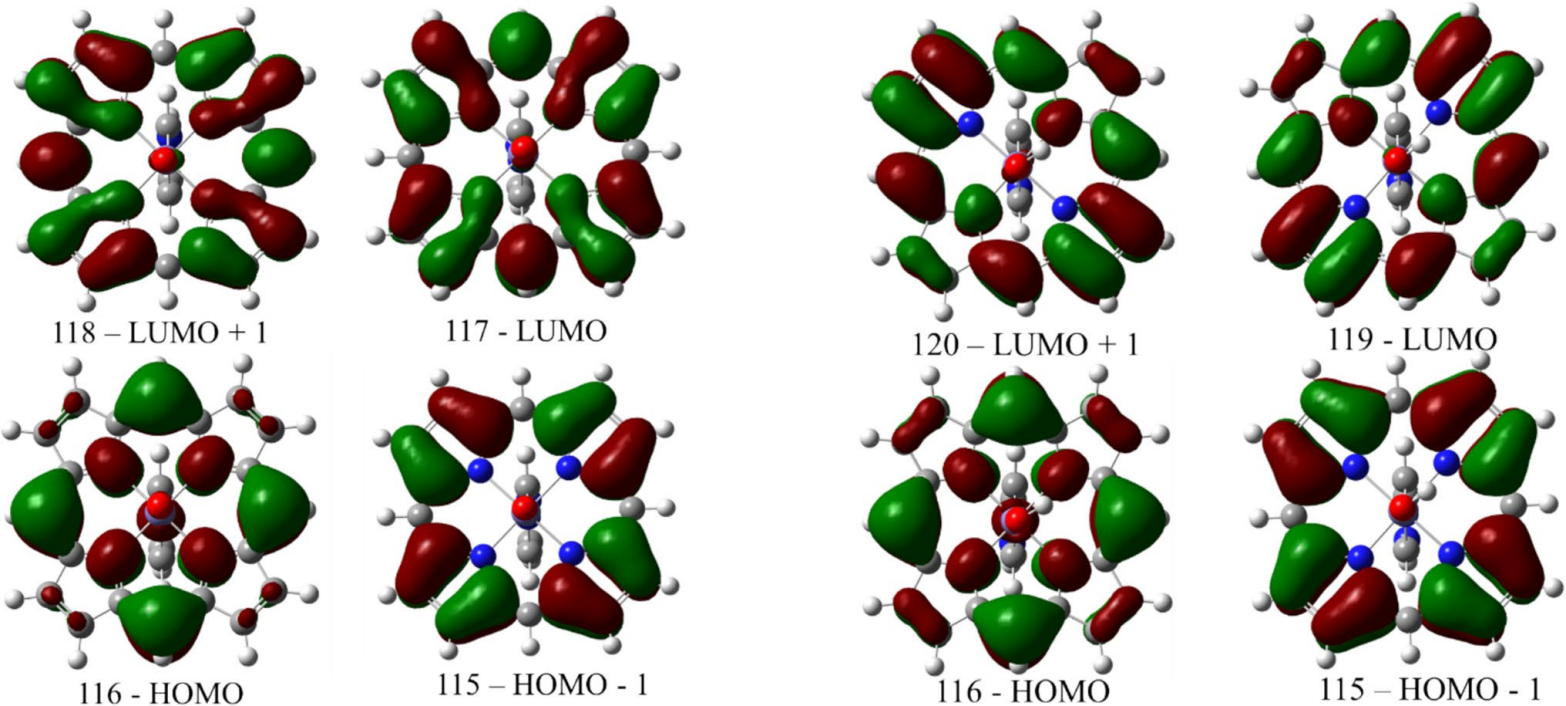
Relevant frontier orbitals of Fe(IV)=O (left) and Fe(IV)–OH (right) imidazole-ligated hemes. From B3PW91/def2-SV(P) geometry optimization

The visible-region elements of the UV–Vis spectra of heme systems are often understood to be easily rationalized within the framework of the Gouterman 4-orbital model; this model has also been applied to metalloporphyrins in conjunction with DFT calculations [41]. Table 1 and Fig. 4 illustrate the energies of the four orbitals responsible for the heme absorption bands in the visible spectrum—the Soret band at ~ 400 nm and the α and β bands at 500–600 nm, for a set of reference systems and then for ferryl vs. protonated ferryl heme. For the ferryl state (unprotonated), several versions of models incorporating non-covalent interactions are also included. It is particularly important to note that the changes in relative orbital energies brought about by these non-covalent interactions (grey cells in Table 1) are of the same order of magnitude as the changes between the experimental states met-aqua, met-hydroxo, and met-cyano—states which, as also indicated in Table 1, do differ dramatically in their UV–Vis spectra. The DFT data thus appear to suggest that slight changes in non-covalent interactions with the oxo-atom in the Mb ferryl would have significant effects on the UV–Vis spectra. Although not shown in Table 1, similar changes are predicted for the elongation of the Fe(IV)-oxo/hydroxo bond (e.g., by ~ 0.1 Å), or for the solvation vs. vacuum. However, the *experimental* data in Figs. 2 and 3 suggests that such changes in the non-covalent environment of the ferryl (or indeed of other states) should have *negligible* effect on the UV–Vis spectra. The apparent difficulty of the DFT methods to predict the effects of non-covalent interactions on the relative energies of the underlying orbitals for the heme systems is not unexpected. Indeed, DFT methods are well known to have limitations when treating very weak interactions [6, 38, 42]. The answer to these shortcomings is usually the calibration using a very narrow set of compounds [8, 43–48]. This, however, would not be a feasible option in our case, considering the expectedly different electronic structures of protonated ferryls vs. non-protonated ferryls.

**Table 1:** The relative energies (in hartree) of the frontier orbitals of the heme (iron oxidation states 3 + and 4 +) with various ligands and with various hydrogen bond partners

Species	Relative Energies (eV)					Experimental (nm)		
	a_2u_ (HOMO-1)	a_1u_ (HOMO)	e_gx_ (LUMO)	e_gy_ (LUMO + 1)	e_gx_-a_1u_	Soret	α	β
Fe(III)–OH_2_	0	0.125	3.088	3.099	2.963	405	500	630
Fe(III)–CN^−^	0	0.171	3.118	3.127	2.947	*419*	540	540
Fe(III)–OH^−^	0	0.174	3.113	3.140	2.939	*410*	542	582
Fe(IV)=O^2−^	0	0.122	3.110	3.127	2.988	*425*	548	584
Fe(IV)=O^2−^–CH_4_	0	0.122	3.110	3.127	2.988			
Fe(IV)=O^2−^–H_2_O	0	0.131	3.102	3.137	2.971			
Fe(IV)=O^2−^–NH_3_	0	0.147	3.083	3.113	2.936			
Fe(IV)=O^2−^–NH_4_^+^	0	0.117	3.121	3.135	3.004			
Fe(IV)-OH	0	0.076	2.071	2.482	1.995	*422*	530	554

## Conclusion

Ferryl Mb undergoes a protonation event below pH 5, as assessed using pH jump experiments with stopped-flow UV–vis spectroscopy. This event is characterized by hypsochromic shifts in the Soret band (~ 5 nm) as well as in the α and β bands (~ 20 to 40 nm) and a ~ 10-nm reduction in the energy difference between the α and β bands. No such shifts are seen upon the denaturation of Mb with guanidine—suggesting that these changes are not caused by changes in solvent exposure or in hydrogen bonding around the ferryl unit. The control experiments with ferric-aqua and ferrous-oxy Mb under the same denaturing conditions (pH jump below pH 5, or guanidine) show that for these states as well denaturation (hence, implicitly, changes in solvent accessibility as well as in hydrogen bonding patterns) do not lead to spectral changes of the order seen in the ferryl pH jump experiments. Together, these data suggest that the protonation event in these latter experiments is localized on the iron-bound oxygen atom, as opposed to somewhere on a hydrogen-bonding partner. TD-DFT calculations are unable to predict the UV–Vis spectra of the heme to the level of detail needed to interpret the experimental findings in this study—i.e., to predict, even semiqualitatively, the existence of the Soret, α, and β bands in a meaningful manner in a set of simple reference models (ferric cyano, hydroxo and aqua, and then compared vs. ferryl and protonated ferryl). Attempts to comparatively rationalize the spectra of ferryl oxo vs hydroxo heme by simply analyzing the energies of the four Gouterman orbitals also suggest a failure of TD-DFT to consistently reflect/depict the effects of subtle structural/environment changes on the set of heme complexes examined in the present study. To conclude, the spectroscopic experimental data presented herein support an observable protonation of the iron-bound oxygen atom in ferryl myoglobin below pH 5.

## Supplementary Information

Below is the link to the electronic supplementary material.

Supplementary file 1 (PDF 1061 kb)

## Data Availability

Data employed for the present article are available in the body of the article and as Supplementary Information. Further details are also available from the authors upon request.
