# Supplementary material for: Globin ferryl species: what is the nature of the protonation event at pH < 5?
Source: J Biol Inorg Chem. 2024 Dec 19;30(1):61–70. doi: 10.1007/s00775-024-02089-3 (PMC11914356; doi:10.1007/s00775-024-02089-3)
Supplement: Supplementary file 1 — (PDF 1061 kb) [file 775_2024_2089_MOESM1_ESM.pdf]

Supporting Information

Globin ferryl species: what is the nature of the protonation event below pH 5?

Cezara Zagrean-Tuza, Lavinia Padurean, Maria Lehene, Adrian M.V. Branzanic, Radu Silaghi-Dumitrescu

Babes-Bolyai University, 11 Arany Janos str, Cluj-Napoca 400028, Romania,

radu.silaghi@ubbcluj.ro, Tel. +40264593833

Benchmarking TD-DFT for histidine-ligated heme systems

Reliable simulation of the UV-vis spectra of hemoprotein spectra using TD-DFT and related calculations has been reported for decades [1–5], and may therefore be expected to be useful for differentiating between the UV-vis spectra of protonated vs. non-protonated ferryl units. To explore this, we analyzed a set of reference systems, starting with the S=5/2 met (ferric) aqua form of histidine-ligated heme, relevant for globins and other histidine-ligated hemoproteins. For this model, a number of functionals and solvation models were tested as shown in Figure S1. The starting point of these trials was a B3PW91 TD calculation at a B3LYP-optimized geometry, as we have recently found that this approach gave the best fit to experiment in a related set of difficult systems, namely the aqua, hydroxo, cyano and peroxo complexes of Co(III) cobalamin.[6] As seen in Figure S1, most of the functionals were able to predict a Soret band close to 400 nm for the S=5/2 ferric-aqua heme model, but always ~50 nm (~0.4 eV) lower than experiment. The best-performing functional was M06-2x/def2SVP, although even in this case agreement with experiment was only semi-quantitative, as the Soret and β bands (the two most intense in the visible region of the spectrum) are both predicted to be shifted by ~50 nm compared to experiment. Other functionals tested for TD-DFT simulations of the met-aqua high-spin model were UB3PW91 (vacuum as well as solvent), UAPFD, M06L, TPSS – with def2-SVP as well as with def2-TZVP basis sets as shown in Figure S1. A number of other functionals were not tested as they yielded significantly meaningless results on related states of the heme. Figure S1 shows that the only functional able to simulate some reasonable semblance of the α and β bands is M06-2x; the next best choices, TPSS and M06L, predict a single band in the 500-600 nm region, instead of the two well-separated bands observed experimentally.[7] None of the other functionals/methods predict

a band of any sort reminiscent of the α and β. The success of the M06-2X functional may be taken to imply a disproportionate role of non-covalent interactions in the small models employed here. Taking into account Figure S1, the M06-2X/def2-SVP approach was subsequently employed for monitoring the effect of axial ligand distortions and of dielectric effects on the spectrum of the ferric-aqua heme.

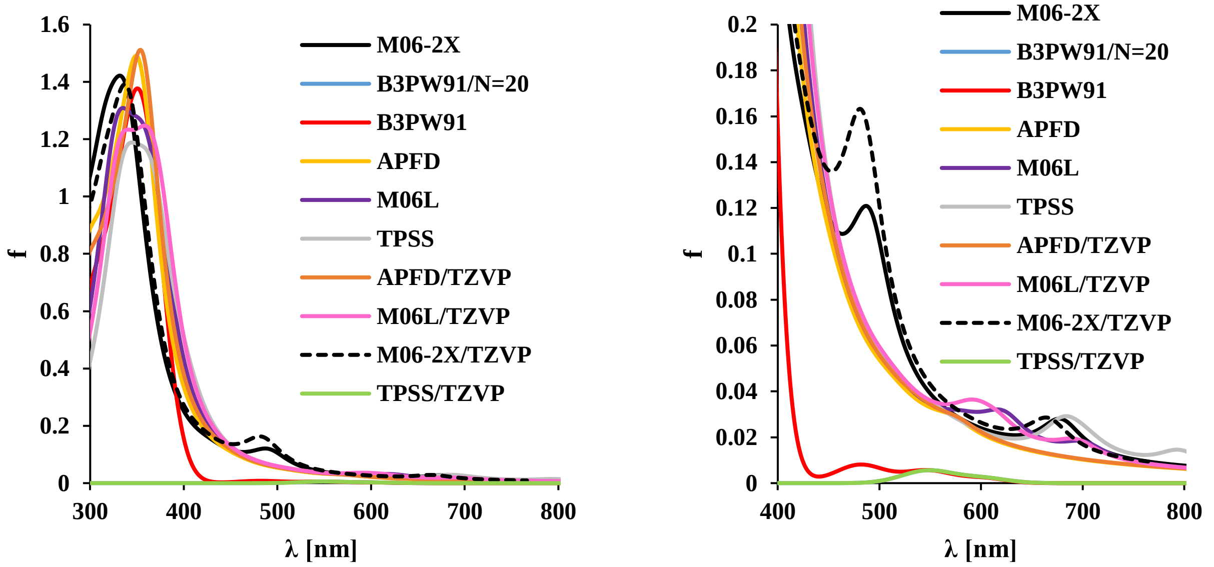

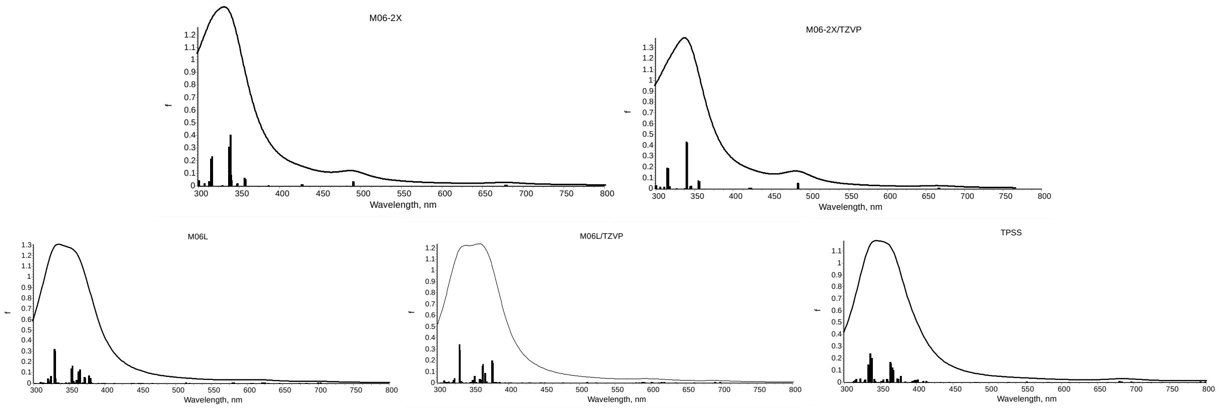

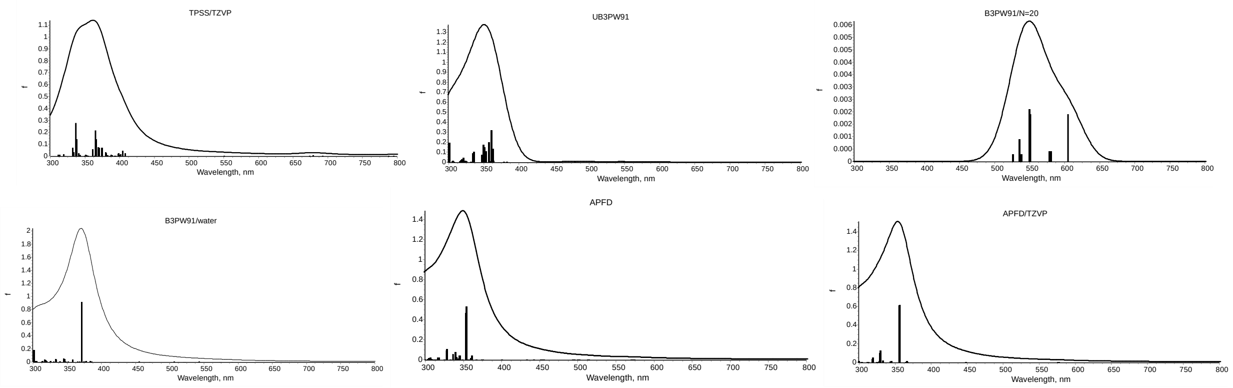

Figure S1. Performance of various functionals for predicting the S=5/2 met-aqua spectrum of a histidine-ligated heme. The def2-SVP basis set in vacuum was employed, unless otherwise specified. The lower rows show individual spectra including representations of the exact positions of the transitions, overlayed under the envelopes shown here. A number of 100 excitations was chosen (and is the default in all results shown here, unless otherwise specified - e.g. N=5, 6, 20 or 200 were also tested).

Figure S2 shows the M06-2X spectra of the S=5/2 ferric-aqua model solvated in media of the dielectric constant from ~80 (corresponding to water) to ~4 (corresponding to diethyl ether, and similar to the interior of a protein). Although solvation does switch the Soret band by ~20 nm (~0.2 eV) compared to vacuum, variation of the dielectric constant in the 4-80 range has no notable effect on the predicted UV-vis spectra.

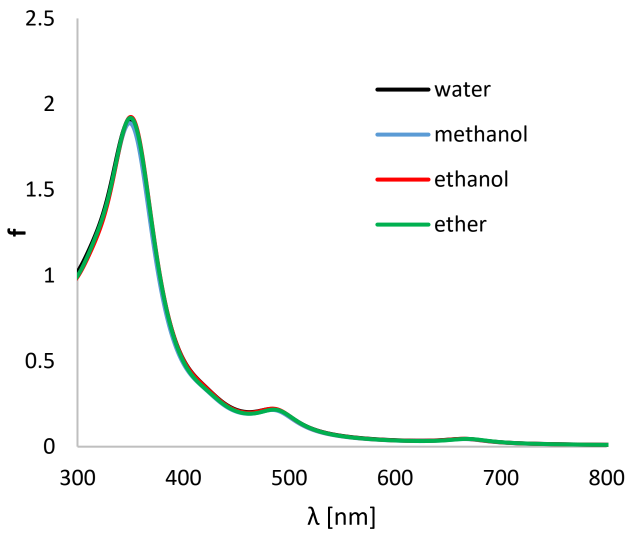

Figure S2. TD-DFT predicted spectra of S=5/2 met-aqua imidazole-ligated heme in various solvents (M06-2X/def2-SVP). See also Figure S4 for individual spectra.

Distortions on the axial ligands (water or imidazole) were further explored cf. Figure S3. Rotation of the ligands or lateral displacement appears to have no notable effects. Elongation of the axial ligands leads to hypsochromic and hyperchromic shifts in the Soret band, and the opposite effect in the α and β bands; also notably, the energy/wavelength difference between these two latter bands does not change significantly. None of these changes match those seen experimentally in ferryl Mb upon protonation below pH 5 (i.e., hypsochromic shifts in all three bands, and a smaller energy difference between the α and β bands).

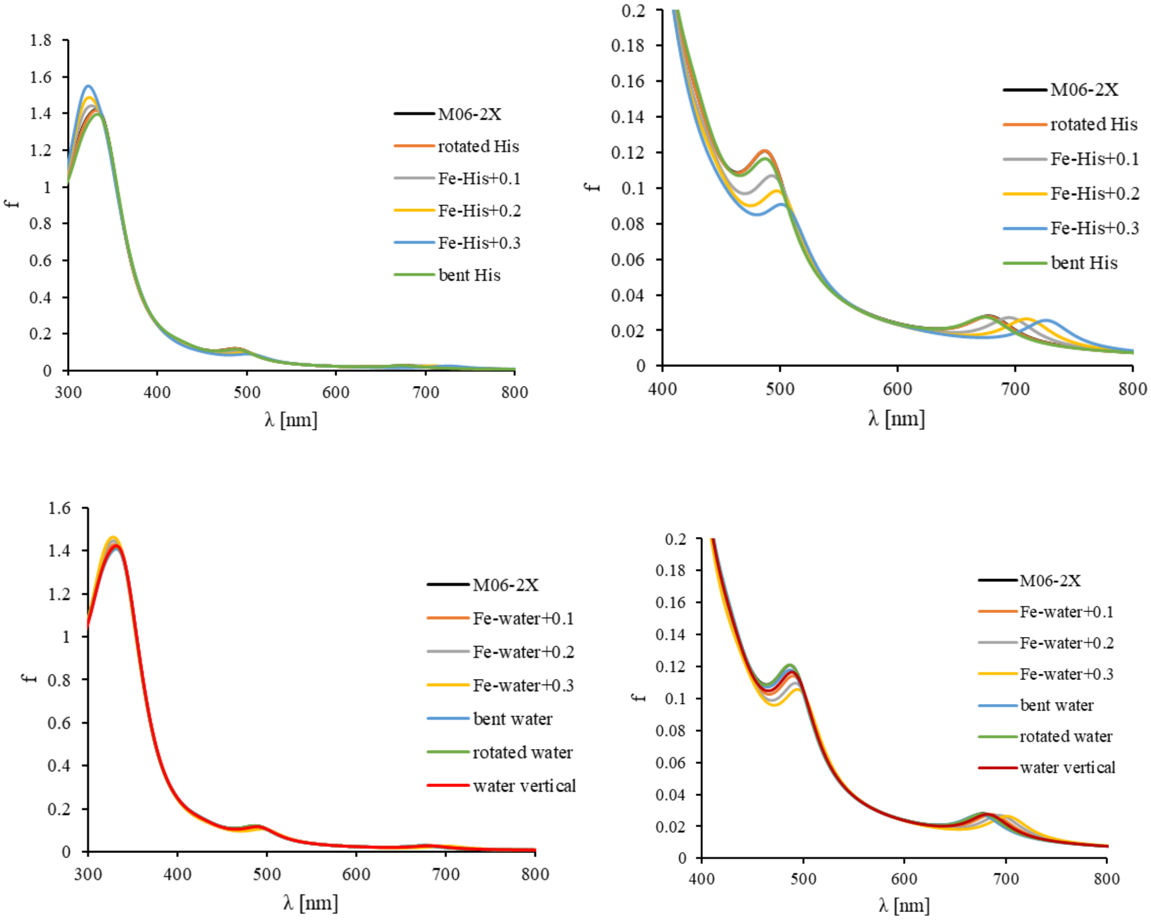

Figure S3. TD-DFT data illustrating the effects of geometrical distortions along the axial ligands. Rotated His: imidazole rotated by 90° around the Fe-N bond/axis; rotated water: water rotated by 45° around the Fe-O bond/axis; bent imidazole/water: water/imidazole moved laterally so that the Fe-N or Fe-O bond forms a 15° angle to the normal onto the heme plane; water vertical: water rotated so that the hydrogen atoms are in the same plane as the oxygen and the iron, pointing upwards; "+0.1/0.2/0.3": Fe-water or Fe-imidazole elongated by 0.1/0.2/0.3 Å. Se also Figure S4 for individual spectra.

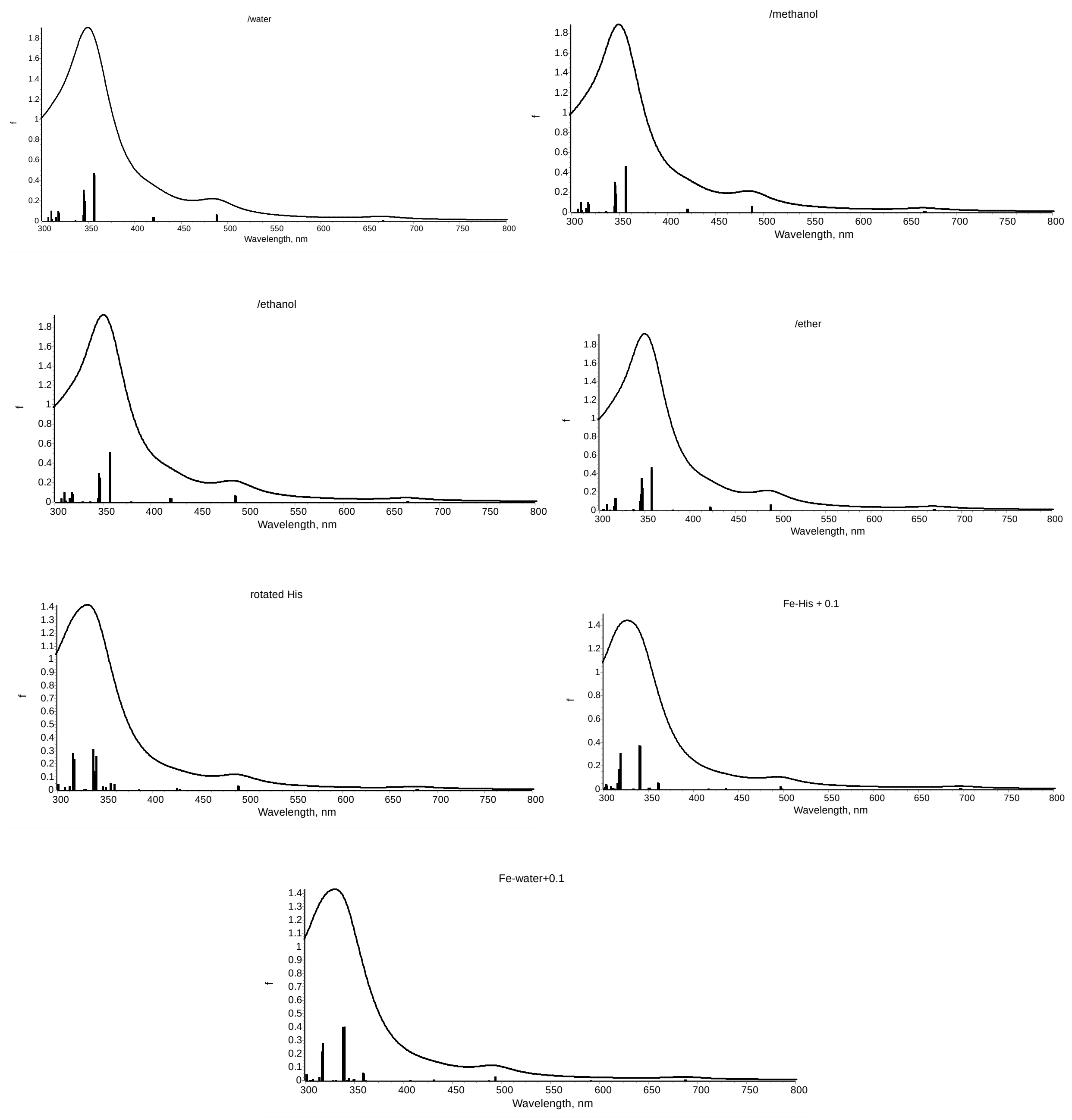

Figure S4. TD-DFT predicted spectra of S=5/2 met-aqua imidazole-ligated heme under various conditions/distortions (M06-2X/def2-SVP).

The data in Figure S1 suggests that the M06-2X would be the only functional to predict a heme spectrum that would include 3 bands in the visible region similarly to experiment for a model of the S=5/2 ferric-aqua state of globins (though still ~50 nm off from the experiment (~0.4 eV for the Soret band, ~0.2 eV for the 630 band)). As shown in Figure S5, the very same methodology

fails on a model of the S=1/2 ferric cyano complex of globins: no α or β bands are predicted at all. Figure S5 also illustrates a dramatic effect of the excitation window (with the smaller excitation window, which should in principle be able to best capture the simple four-orbital Gouterman model, failing to now even predict a Soret band at all) and on the solvent. Figure S6 shows that the same problems arise for the related S=1/2 ferric-hydroxo model. In this case, the CAMB3LYP method was also employed, again with no improvement over B3PW91.

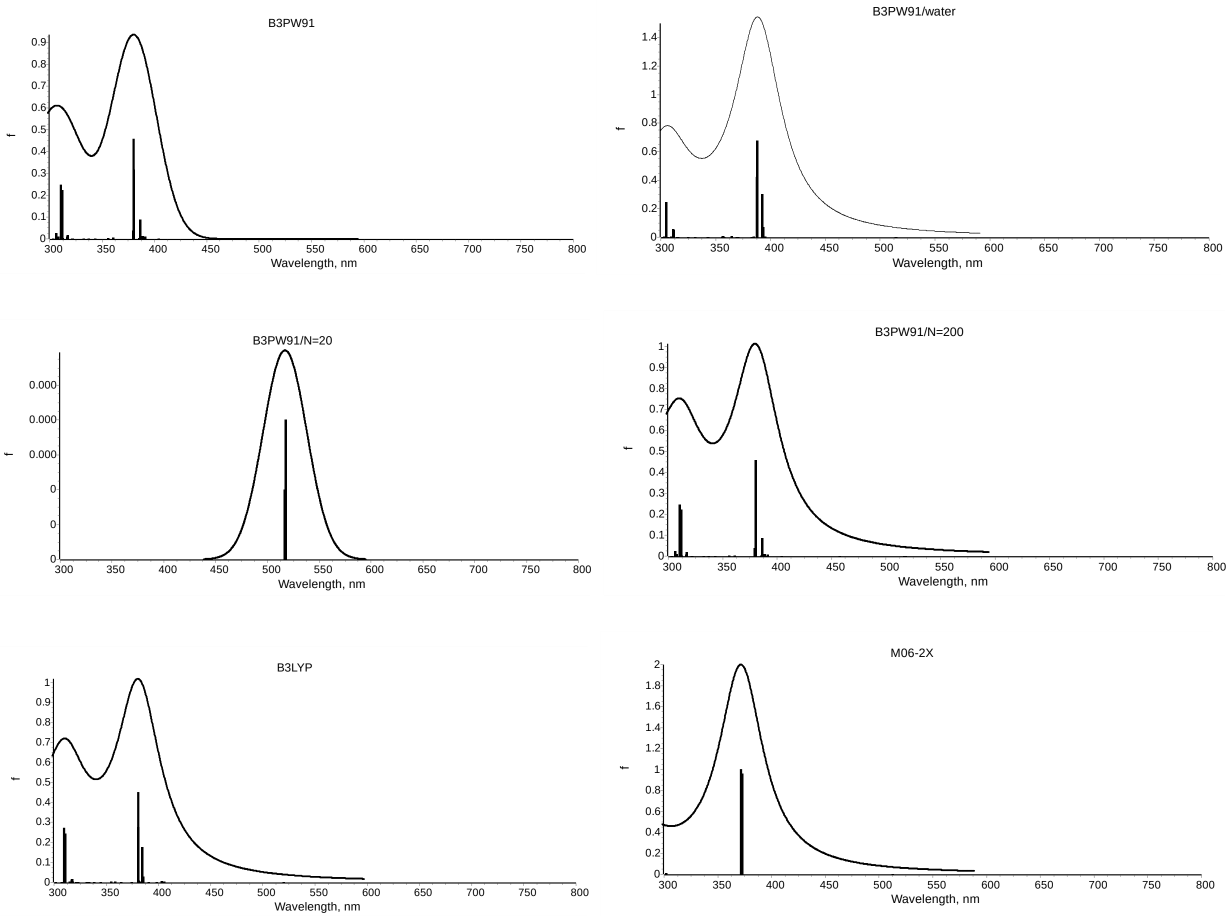

Figure S5. UV-vis spectra from TD-DFT calculations with various methodologies for S=1/2 ferric-cyano imidazole-ligated heme.

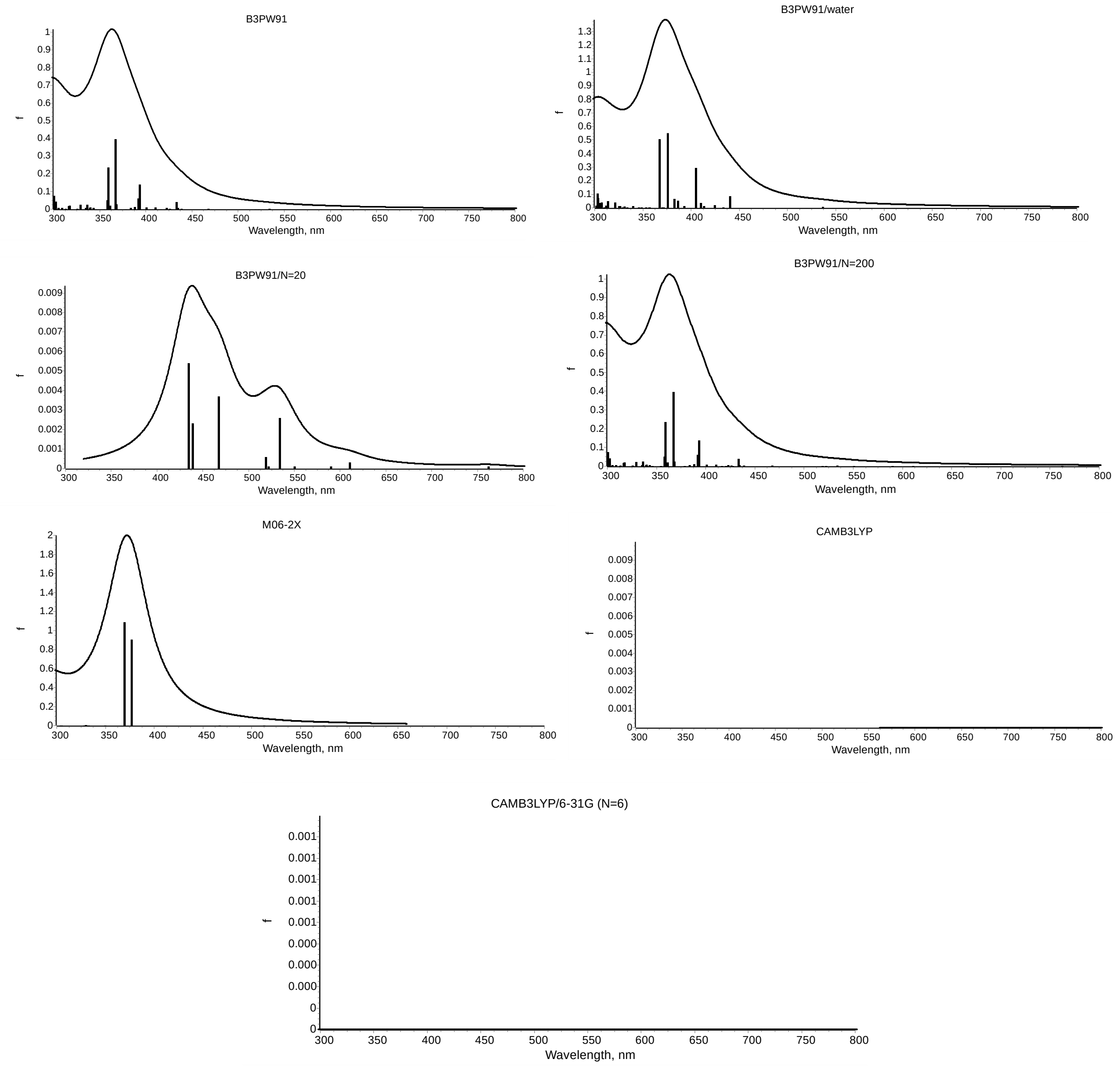

Figure S6. UV-vis spectra from TD-DFT calculations with various methodologies for S=1/2 ferric-hydroxo imidazole-ligated heme.

Together, the tests on the three ferric reference models, aqua, cyano, and hydroxo do in fact suggest that not one single TD-DFT method can qualitatively predict UV-vis spectra of heme complexes in general – and that any results bearing semblance to experiment can be obtained only by cherry-picking a single model or a very narrow set of models. Indeed, here, the S=1/2 hydroxo and cyano ferric heme-histidine models behave very similarly to each other – but then very

different to the S=1/2 ferric aqua; apparently, a change in spin state and/or overall charge /protonation state is enough to change the performance of a functional dramatically. Figures S4-S6 show that the Fe(IV)-oxo and Fe(IV)-hydroxo models suffer from the same problems as the S=1/2 models, in that the α and the β bands cannot be modeled in a meaningful comparative way between the two protonation states. Figure S8 also shows that inclusion of elements that reduce porphyrin symmetry (i.e., lateral substituents, and even structural distortions from planarity copied from the crystal structure) do not lead to predicted TD-DFT spectra that would include the α and the β bands in both the Fe(IV)-oxo and Fe(IV)-hydroxo models.

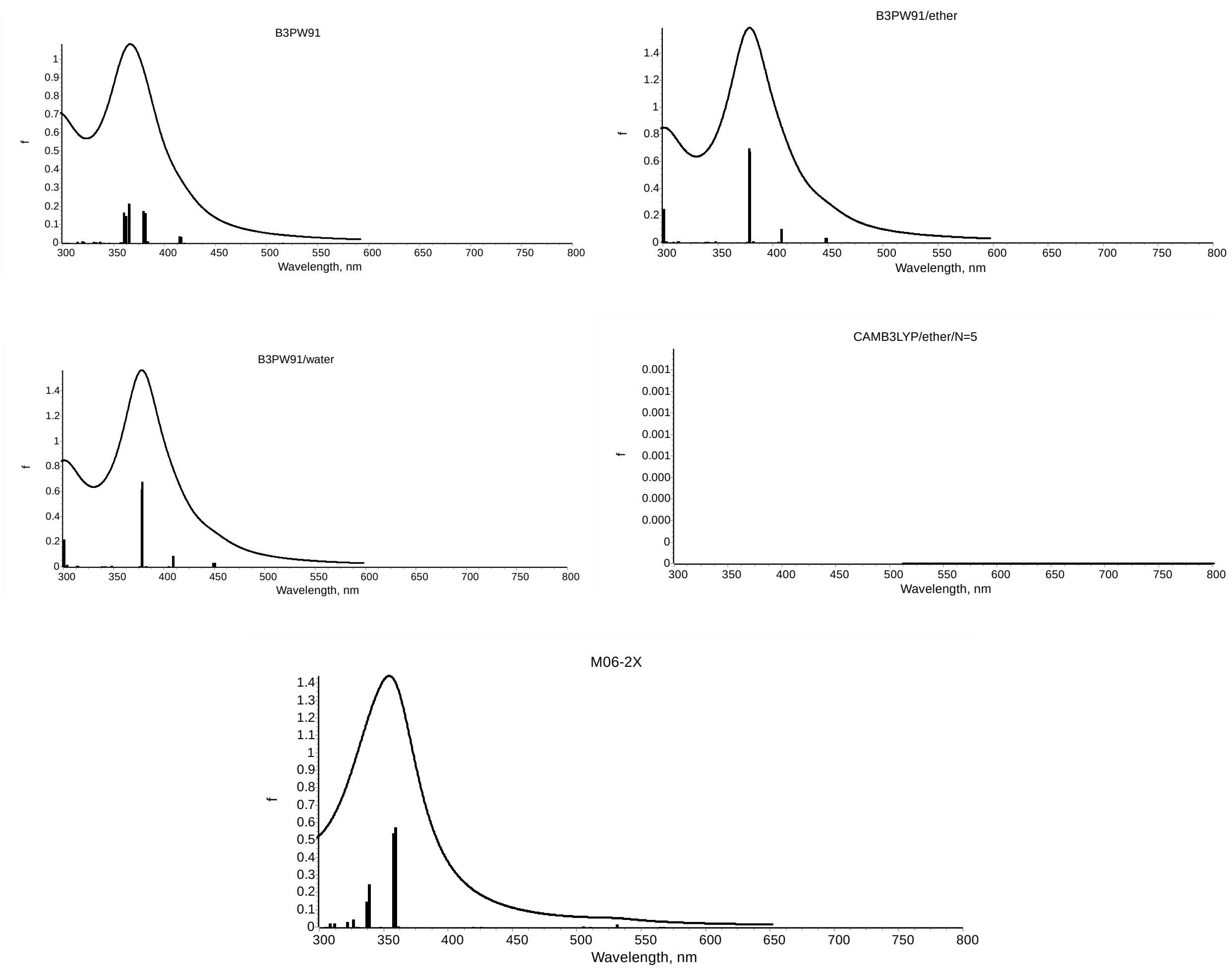

Figure S7. UV-vis spectra from TD-DFT calculations with various methodologies for S=1 Fe(IV)-oxo imidazole-ligated heme.

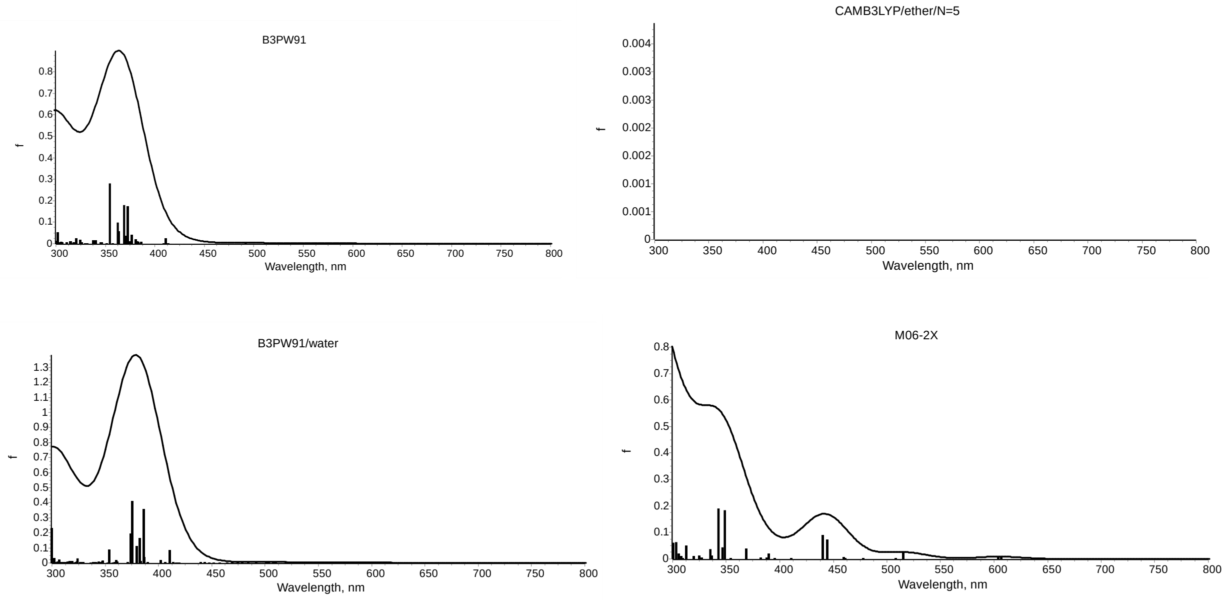

Figure S8. UV-vis spectra from TD-DFT calculations with various methodologies for S=1 Fe(IV)-hydroxo imidazole-ligated heme.

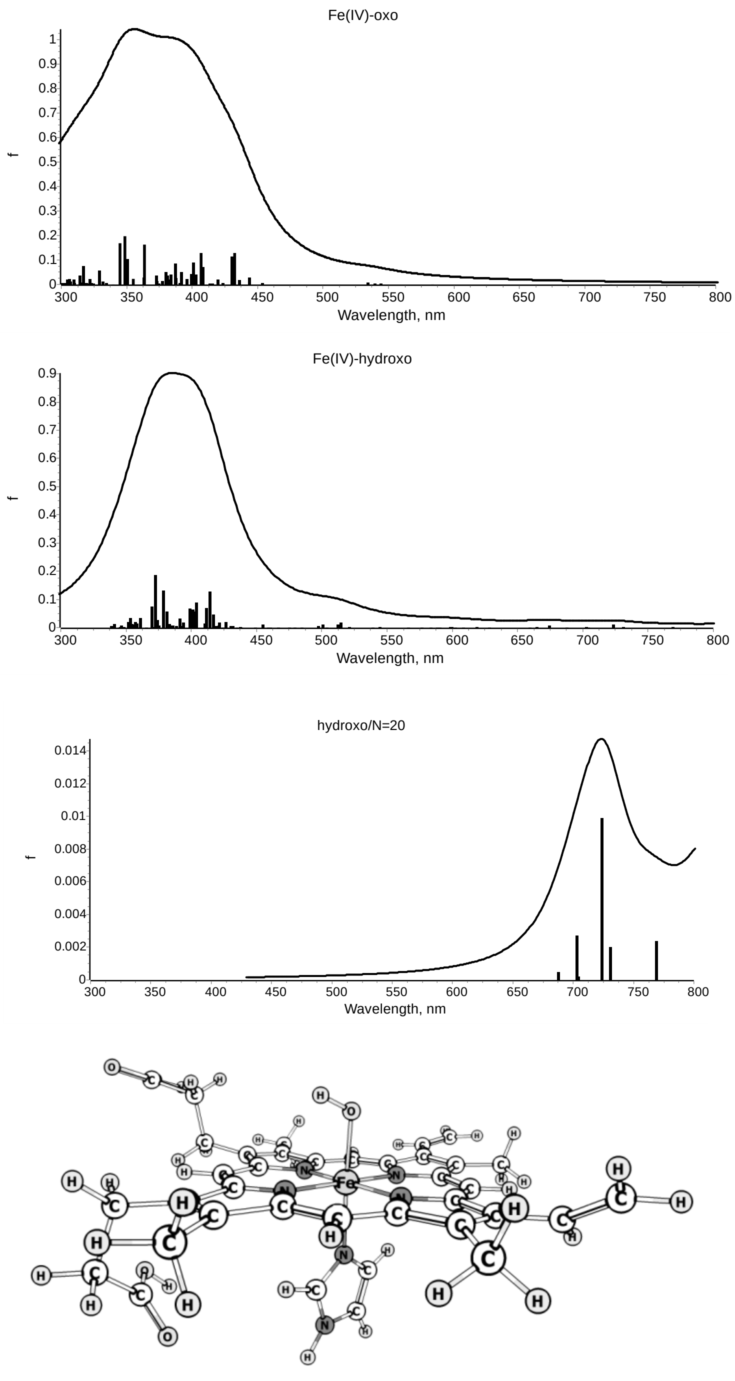

Figure S9. UV-vis spectra from TD-DFT calculations for S=1 Fe(IV)-oxo and Fe(IV)-hydroxo imidazole-ligated heme, including the lateral substituents at the heme and freezing the structure at coordinates seen in the myoglobin Fe(IV)-hydroxo crystal structure of Hersleth et al.[8] The oxo model was identical to the hydroxo, except for the oxygen-bound proton and for the Fe-O distance, which was set to 1.66 Å. The carboxylate groups were modeled as neutral (protonated) carboxyl, in order not to artificially raise the charge of the model to too negative values, which would make the results less physically meaningful. Lower row: structure of the hydroxo model employed in these calculations.

References:

1. Loew GH, Harris DL (2000) Role of the heme active site and protein environment in structure, spectra, and function of the cytochrome P450s. Chem Rev 100:407–419

2. Attia AAA, Cioloboc D, Lupan A, Silaghi-Dumitrescu R (2016) Multiconfigurational and DFT analyses of the electromeric formulation and UV–vis absorption spectra of the superoxide adduct of ferrous superoxide reductase. J Inorg Biochem 165:49–53. https://doi.org/10.1016/j.jinorgbio.2016.09.017

3. Silaghi-Dumitrescu R (2011) What causes iron-sulphur bonds in active sites of one-iron superoxide reductase and two-iron superoxide reductase to differ? Chem Pap 65:559–565. https://doi.org/10.2478/s11696-011-0023-4

4. Lupan A, Matyas C, Mot A, Silaghi-Dumitrescu R (2011) Can geometrical distortions make a laccase change color from blue to yellow? Stud Univ Babes-Bolyai Chem 56:231–238

5. Namuangruk S, Sirithip K, Rattanatwan R, et al (2014) Theoretical investigation of the charge-transfer properties in different meso-linked zinc porphyrins for highly efficient dye-sensitized solar cells. Dalton Trans 43:9166–9176. https://doi.org/10.1039/c4dt00665h

6. Lehene M, Plesa D, Ionescu-Zinca S, et al (2021) Adduct of Aquacobalamin with Hydrogen Peroxide. Inorg Chem 60:12681–12684. https://doi.org/10.1021/acs.inorgchem.1c01483

7. Antonini E, Brunori M (1971) Hemoglobin and Myoglobin in their Reaction with Ligands. North-Holland, Amsterdam

8. Hersleth HP, Dalhus B, Gorbitz CH, Andersson KK (2002) An iron hydroxide moiety in the 1.35 A resolution structure of hydrogen peroxide derived myoglobin compound II at pH 5.2. J Biol Inorg Chem 7:299–304
